# Quantifying network behavior in the rat prefrontal cortex

**DOI:** 10.3389/fncom.2024.1293279

**Published:** 2024-08-29

**Authors:** Congzhou M. Sha, Jian Wang, Richard B. Mailman, Yang Yang, Nikolay V. Dokholyan

**Affiliations:** ^1^Department of Engineering Science and Mechanics, Penn State University, University Park, PA, United States; ^2^Department of Pharmacology, Penn State College of Medicine, Hershey, PA, United States; ^3^Department of Neurology, Penn State College of Medicine, Hershey, PA, United States; ^4^Department of Biochemistry and Molecular Biology, Penn State College of Medicine, Hershey, PA, United States; ^5^Department of Chemistry, Penn State University, University Park, PA, United States; ^6^Department of Biomedical Engineering, Penn State University, University Park, PA, United States

**Keywords:** neurophysiology, dynamic time warping, rat prefrontal cortex, decision-making, non-parametric test, neuron synchronization

## Abstract

The question of how consciousness and behavior arise from neural activity is fundamental to understanding the brain, and to improving the diagnosis and treatment of neurological and psychiatric disorders. There is significant murine and primate literature on how behavior is related to the electrophysiological activity of the medial prefrontal cortex and its role in working memory processes such as planning and decision-making. Existing experimental designs, specifically the rodent spike train and local field potential recordings during the T-maze alternation task, have insufficient statistical power to unravel the complex processes of the prefrontal cortex. We therefore examined the theoretical limitations of such experiments, providing concrete guidelines for robust and reproducible science. To approach these theoretical limits, we applied dynamic time warping and associated statistical tests to data from neuron spike trains and local field potentials. The goal was to quantify neural network synchronicity and the correlation of neuroelectrophysiology with rat behavior. The results show the statistical limitations of existing data, and the fact that making meaningful comparison between dynamic time warping with traditional Fourier and wavelet analysis is impossible until larger and cleaner datasets are available.

## Introduction

### Background

Hodgkin and Huxley’s model of neuron action potentials using the squid giant axon (Hodgkin and Huxley, 1952) was the seminal event in neurophysiology (Coull, 1998; Citron, 2012; Kucyi and Davis, 2017). The central dogma of modern neuroscience is that neuron electrochemical activity and connectivity at the microscopic level can provide a clear understanding of complex behaviors. Thus, measuring electrical signals should be sufficient to integrate the chemical signaling and activity of neural networks.

The visual cortex is one of the better-understood neural networks because there are significant spatial correlations in signals that result from local connections between neurons. These spatial autocorrelations result in a high signal-to-noise ratio due to the strong intensity of the electric field produced by local synchronized neuron firing. Additionally, the neuroelectrophysiology of individual cells in the visual pathway has allowed mathematical modeling of the performance of specific neurons (Hartline, 1969; Gilbert and Wiesel, 1992). The spatial autocorrelations of visual neural activity are sufficiently high such that low-resolution, indirect measurements of neuron activity, such as local blood flow (Miyawaki et al., 2008; Shen et al., 2019; Ren et al., 2021) and extracranial electrodes (Wakita et al., 2021) that record neuron activities in a large scale, are useful to reconstruct perceived images. The organization of the visual cortex may be explained by the fact that images projected on the retina are spatially correlated, and therefore biological neural networks have adapted to take advantage of these correlations.

From an evolutionary perspective, the visual cortex is an ancient structure, and therefore the neural networks involved in visual processing have optimized over time. In contrast, the prefrontal cortex as part of the neocortex is one of the least developed brain regions, especially through evolutionary analysis of its size in mammals (Teffer and Semendeferi, 2012; Preuss and Wise, 2022). Unlike the visual cortex, the prefrontal cortex does not seem to possess highly local and regular spatial organization (O’Reilly, 2010). Because of the lack of regular spatial organization of the prefrontal cortex and the consequent low intensity of electrical signals, electrophysiology is one of the only methods with adequate sensitivity to probe the fine structure of the prefrontal neural network. Unfortunately, such measurements have relatively small scale, are highly invasive, and experiments are only feasible on laboratory animals such as rats (Ito et al., 2018; Stout and Griffin, 2020; Yang et al., 2022).

### Identification of the problem

We first hypothesized that there might be theoretical limits of existing experimental methodology regarding the prefrontal neuron firing. We used a simple model of neural circuits to estimate those limits and outlined the statistical considerations for experiment planning in rat neuroelectrophysiology. We then sought to elucidate the relationship between prefrontal neuron firing and behavior based on the currently available resource and tools. To capture key network characteristics from recorded spike trains and local field potentials (LFPs), a myriad of tools has been tried. For example, the dimensionality reduction has been used to unveil the multidimensional dynamic encoding in the prefrontal cortex (Aoi et al., 2020). On the other hand, we believe that the prefrontal network characteristics are fundamentally embodied by the level of synchronicity of neuron firing: two neurons that work in concert will have spike trains and/or LFPs that are highly correlated or anti-correlated in time, whereas two neurons that process independent data or function in separate circuits will not be correlated. Although it also is quite possible for variables to be dependent but uncorrelated. The question is which mathematic and statistic tools should be used to evaluate the neural synchronicity on the data collected through the current existing experimental methodology that has potential limits as mentioned above and in the first part of the discussion section below.

When analyzing complex data such as neuroelectrophysiology tracings, the major statistical concern is bias and overfitting (Cawley and Talbot, 2010). Although large amounts of data are collected in these experiments, the degrees of freedom are severely restricted by the number of animals and recordings collected (Marek et al., 2022). When researchers choose a statistical model for their data, certain assumptions are made about the structure of the data. Commonly, a class of statistical models is chosen with hyperparameters tuned to best fit the data. The more hyperparameters that must be tuned, the fewer degrees of freedom remain for the subsequent goodness-of-fit tests. Some examples of the tuning of various hyperparameters by some classical modeling are described in the following section. In this work, we sought to maximize the degrees of freedom remaining after modeling to address our scientific questions. Schematically, we must allocate the degrees of freedom in the data to the modeling we perform and the scientific questions we seek to answer:


(1) $${dof}_{\mathrm{data}}={dof}_{\mathrm{model}}+{dof}_{\mathrm{science}}$$

The more degrees of freedom we can devote to the scientific question, the more confident we can be in our statistical tests.

### A potential solution

We chose to investigate neuron synchronicity using the dynamic time warping (DTW) method (Vintsyuk, 1972). DTW is an efficient, non-parametric approach to determine the best alignment of two time series, such that the overall shapes of the time series are matched. Compared to classical Fourier and wavelet analysis (Ito et al., 2018; Stout and Griffin, 2020), DTW gives a global alignment of the data that is robust to local variations in timing. We predicted that DTW could discern if two neurons fire synchronously or asynchronously. In contrast, local timing variations in low frequency signals such as firing rate and voltage lead to destructive interference when performing the Fourier or wavelet transform, decreasing the signal-to-noise ratio. In practice, these techniques require the tuning of various hyperparameters to smooth and denoise the data. Examples include a window size for the spike-triggered LFP average (Ito et al., 2018), cutoff frequencies for band-pass filters (Ito et al., 2018), various bootstrapping techniques (Ito et al., 2018), and choice of kernel for support vector machines (Stout and Griffin, 2020). In contrast, traditional DTW uses no additional parameters to yield a dissimilarity index for each pair of spike trains or LFP tracings, therefore retaining more degrees of freedom for answering statistical questions. Though nonparametric techniques (model free) in general will have lower power comparing to parametric techniques (model based), the penalty in the latter is model misspecification.

Thus, we analyzed electrophysiology data collected from rats performing the T-maze task, a task that evaluates working memory. We use previously published spike train and LFP recordings taken from the rat medial prefrontal cortex (mPFC) (Ito et al., 2018; Stout and Griffin, 2020; Yang et al., 2022), along with previously unpublished data from three additional rats (rats A, B, and C) recorded using published methods (Yang et al., 2022). We investigated if DTW would be useful in elucidating the connection between neural activity and behavior (Results), and we examined the underlying assumptions of neuroelectrophysiology experiments (Discussion). This study, by using DTW as a representative technique and the rat spike train and LFP recordings during the T-maze alternation task as a representative protocol, provided a concrete and substantial, technical discussion on the scientific and reproducibility issues faced by all rat PFC researchers.

## Results

**Figure 1:**
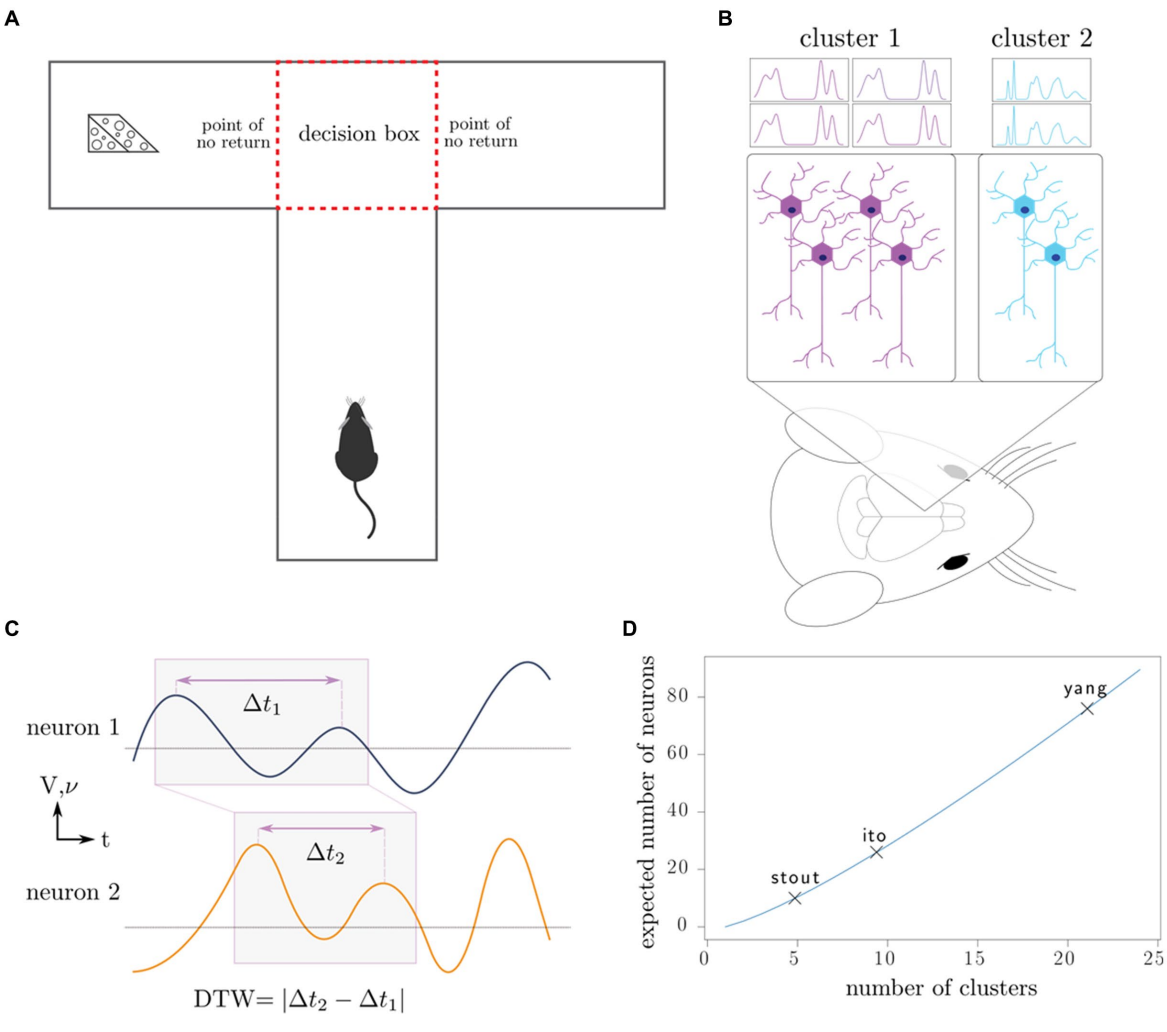
Outline of experiments, our working model, and data analysis. **(A)** Rats complete the T-maze task, in which the reward switches locations after each completion of the task. This task tests the working memory and decision-making capacity of the rat. **(B)** Neurons in the medial prefrontal cortex are recorded using intracranial electrodes, resulting in the measurement of specific clusters of neurons. **(C)** Dynamic time warping computes an optimal alignment of neuron spike trains (frequency 𝝂) and local field potentials (voltage V), producing a single number characterizing the synchronicity of the neurons. **(D)** Depending on the number of distinct neuron clusters in the region of interest, a certain number of neurons must be sampled to produce a representative sample of all the clusters; in combinatorics, this is known as the coupon collector problem. We plot the number of clusters that the maximum number of neurons recorded in each of the three studies can theoretically discern.

Our theoretical arguments for the low statistical power of existing experimental designs are presented in the Discussion and Methods (*Reproducibility of electrode recordings*). The data from the three published studies (Table 1) had rat neuron spike trains and/or LFPs recorded as the rats performed in T-maze (Figure 1A). The exact experimental details for the three studies differed slightly (Ito et al., 2018; Stout and Griffin, 2020; Yang et al., 2022), but their combined scientific goal was to analyze the link between neuron recordings and working memory performance in the T-maze task. We focused on the four-second window of time centered on the moment at which the rat leaves the T-intersection (decision box in Figure 1A), presumably making its choice. This window contained 2 s of pre-decision neural activity (e.g., cognition and decision-making process) and 2 s of post-decision neural activity (e.g., evaluation of reward or lack of reward). Since the experimental designs differed among the studies [e.g., the rat either traversed the T-maze continuously (Ito et al., 2018; Stout and Griffin, 2020) or was picked up by the researcher in between traversals (Yang et al., 2022)], we used this limited window of time for our analysis to limit the effects of the design, constituting one of the only hyperparameters chosen in our analysis. Our hypothesis was that neural activity was affected significantly by the independent variables of (1) timing of the recording relative to the choice, (2) correctness of the choice, and (3) sampled neurons (Table 2). Null and alternative hypotheses for each statistical test we performed are included in the Supplemental Tables S1–S3.

**Table 1:** Characteristics of the three studies analyzed in this work.

	Ito et al. (2018)	Stout and Griffin (2020)	Yang et al. (2022)
Year of publication	2018	2020	2022
Number of rats	3	5	9
Total number of sessions (including sessions in which only one neuron was recorded)	13	45	13
Sessions excluded (sessions in which only a single neuron was recorded)	0	9	0
Number of trials (excluding single-neuron recordings)	790	1,508	472
Mean neurons recorded (s.d.)	17.56 (4.24)	3.31 (2.21)	31.8 (28.55)
Range of neurons recorded	[10, 26]	[1, 10]	[5, 76]
Total neurons recorded	232	89	314
Fixed electrode	No	No	Yes
Spatial metadata	Yes	Yes	Yes

**Table 2:** Listing of categorical variables used for stratification/grouping in our analyses.

Categorical variable	Values	Interpretation
Timing	Before, after	Data from before vs. after the rat left the T-intersection
Correctness	True, false	True if rat chose the correct (i.e., alternate) branch of the T-maze during the task
Study	Ito, Stout, Yang	The study to which the rat belongs
Rat	17 total animals	The rat identity
Session	62 total sessions (with more than 1 neuron recorded)	The rat identity and the date of recording

**Figure 2:**
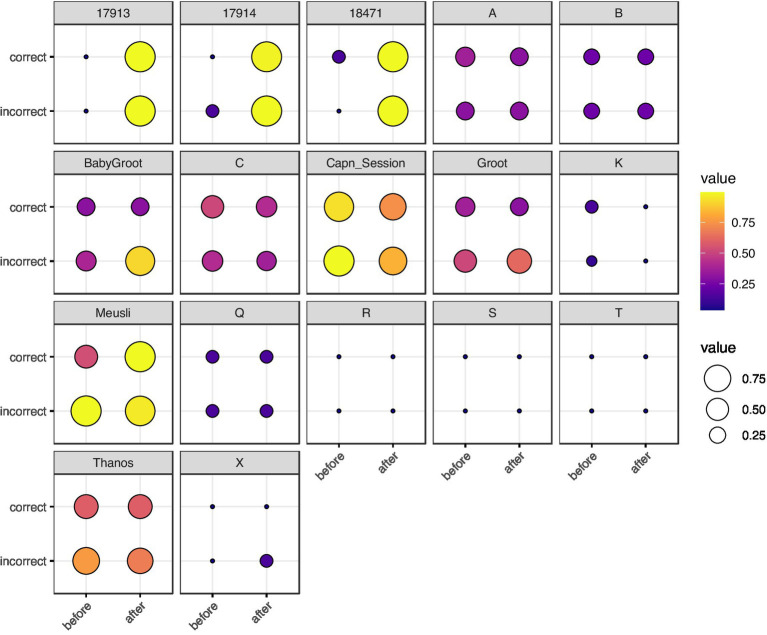
Balloon plot of neuron spike train $d_{\mathrm{crit}}$ , grouped by rat, correctness of T-arm choice, and timing with respect to T-arm choice. Each box represents a single rat. The x-axis represents timing (before vs. after the rat visits the T-maze intersection) and the y-axis represents correctness of T-arm taking (True vs. False). The size and color of each marker represents $\log_{10}d_{\mathrm{crit}}$ . For rats with multiple trials, we took the mean of $\log_{10}d_{\mathrm{crit}}$ . Rats 17,913, 17,914, and 18,471 are from Ito et al. (2018); single letter rats are from Yang et al. (2022); the remaining five rats are from Stout and Griffin (2020). The most visually striking difference is in Ito et al. (2018) rats, in which there is a stark contrast for spike trains recorded before vs. after making a choice.

We used the DTW method on the neuron spike trains from all three studies and from the local field potentials for Ito et al. (2018) and Yang et al. (2022) (Figures 1B,C). Since DTW provides a numerical measurement of dissimilarity between firing of neurons, we converted the resulting DTW matrices into undirected, unweighted graphs (Supplemental methods). For each set of neuron spike trains and/or LFPs, we quantified the connectivity of the resulting DTW graph by a single number, *d*_crit_ (Methods). To evaluate for the presence of differences among sets of experiments, we used non-parametric statistical tests (Kruskal-Wallis, Kolmogorov–Smirnov, Mantel, and Boschloo exact tests) to mitigate both our lack of knowledge of the true underlying probability distributions and the small sample sizes. These statistical tests are described in detail in the Supplemental methods. We visualized $d_{\mathrm{crit}}$ as a function of our independent variables in Figure 2 (spike trains) and Supplementary Figure S1 (LFPs).

We defined a *trial* as a single traversal of the T-maze by a rat. We defined a *session* as all trials recorded on a single day for a single rat. In the Ito et al. (2018) and Stout and Griffin (2020) studies, electrodes were adjusted between sessions, and, therefore, a different set of neurons were recorded between sessions. We set the level of statistical significance (false positive rate) to α = 0.05.

### Neural network firing is consistent across a single session

Our first question was whether we could pool trials from a single session in analyzing *d*_crit_? The null hypothesis was that during a single session, we may assume that the rat basal activity is unchanged between recordings in the same session, as measured by *d*_crit_, The alternative hypothesis is that specific factors affect *d*_crit_ between trials in the same session, such as the categorical variables in Table 2.

First, we analyzed the normality of *d*_crit_ with the Shapiro–Wilk test (Supplementary Tables S1, S2). For neuron spike trains, we found that in 96.7% (60 out of 62) of sessions, the samples were not normally-distributed (*p* < 0.05). For LFPs, we found that 80.0% (20 out of 25) were not normally-distributed. Thus, parametric tests are inappropriate. Comparing spike trains to LFP with Boschloo’s exact test, these fractions were not significantly different (*p* = 0.615). We performed subgroup analysis on the categorical variables (1) correctness and (2) timing and found that this proportion was unaffected by further stratification by these variables. Therefore, we concluded that the Kruskal-Wallis non-parametric test was more appropriate than classical one-way analysis of variance (ANOVA), due to violation of the assumption of normally-distributed data and due to the limited number of trials per session.

Only 4.8% (3 out of 62) of the spike train sessions displayed significant (Kruskal-Wallis *p* < 0.05) trial-to-trial variance of *d*_crit_, regardless of stratification by correctness and/or timing; the corresponding proportion for LFPs was 12% (3 out of 25). For completeness, we also performed ANOVA and found that 11.2% (7 out of 62) of the spike train sessions displayed significant trial-to-trial variance, regardless of stratification; the corresponding proportion for LFPs was 20% (5 out of 25). We tested if Kruskal-Wallis gave different results as compared to ANOVA using Boschloo’s exact test, and found that neither the spike train data (*p* = 0.238), nor the LFPs (*p* = 0.378), were significantly different.

Since few of the sessions (within our 5% margin of error for false positives from α = 0.05) had a significant trial-to-trial variance in the spike trains, we concluded that we may perform pooled analysis of *d*_crit_ for the spike trains of trials across a single session. Boschloo’s exact test on the LFPs also demonstrated a non-significant deviation from the 5% margin of error (*p* = 0.413 for Kruskal-Wallis, *p* = 0.140 for ANOVA).

### Neural network differences exist across multiple sessions for a single rat

Our second question was: when analyzing *d*_crit_, can we pool all the trials for a single rat, regardless of the day the recordings were taken? Using the Kruskal-Wallis test, we found that 70% (7 of 10) rats displayed significant (*p* < 0.05) day-to-day variance of *d*_crit_ in their spike trains, regardless of additional stratification by (1) correctness and (2) timing (Supplementary Table S1). The caveat was that we excluded six rats from the Yang study (Yang et al., 2022) in this analysis, because we were unable to apply the Kruskal-Wallis test since recordings for those rats were only performed on a single day (dof = 0). Additionally, the three rats that did not display significant differences across sessions were 17,914 and 18,471 from the Ito study (Ito et al., 2018) and rat A from Yang et al. (2022). The same analysis was performed for LFPs, in which 80% (4 out of 5) rats (all rats except rat A from Yang et al., 2022) displayed significant variance (Supplementary Table S2). For the LFPs, the five rats from Stout and Griffin (2020) were excluded since the LFPs were not recorded for those rats. Boschloo’s exact test showed no difference between spike trains and LFPs (*p* = 0.922). We conclude that the resting cognitive state for each rat may be subject to change depending on the day that the recording is performed.

### Significant neural network heterogeneity exists between rats and studies

Our third question was: does *d*_crit_ depend on the specific rat? Using the Kruskal-Wallis test, we found that the Stout and Griffin (2020) and Yang et al. (2022) studies displayed significant (*p* < 0.001) rat-to-rat differences in their spike trains, whereas Ito et al. (2018) was non-significant (*p* = 0.206). Ito et al. (2018), however also had the fewest rats (*n* = 3) potentially leading to the insufficient statistical power of the Kruskal-Wallis test. The LFPs showed significant (*p* < 0.001) rat-to-rat variation in both Ito et al. (2018) and Yang et al. (2022). Pooling all rats, we confirmed (*p* < 0.001) that the specific study under consideration affects the value of *d*_crit_ for both spike trains and LFPs. We conclude that significant heterogeneities exist among studies, animals, and sessions. The statistical tests supporting this conclusion are summarized in Supplementary Table S1 (spike trains) and Supplementary Table S2 (LFPs).

### DTW distance is inconsistently correlated with physical electrode distance

Do correlations in firing among neurons reflect the underlying spatial distance between the neurons? We used both Welch’s *t*-test for unequal variances and the non-parametric Kolmogorov–Smirnov test (KS-test) to test for differences in the distribution of the DTW distance when grouped by the spatial distance of the associated electrodes.

**Figure 3:**
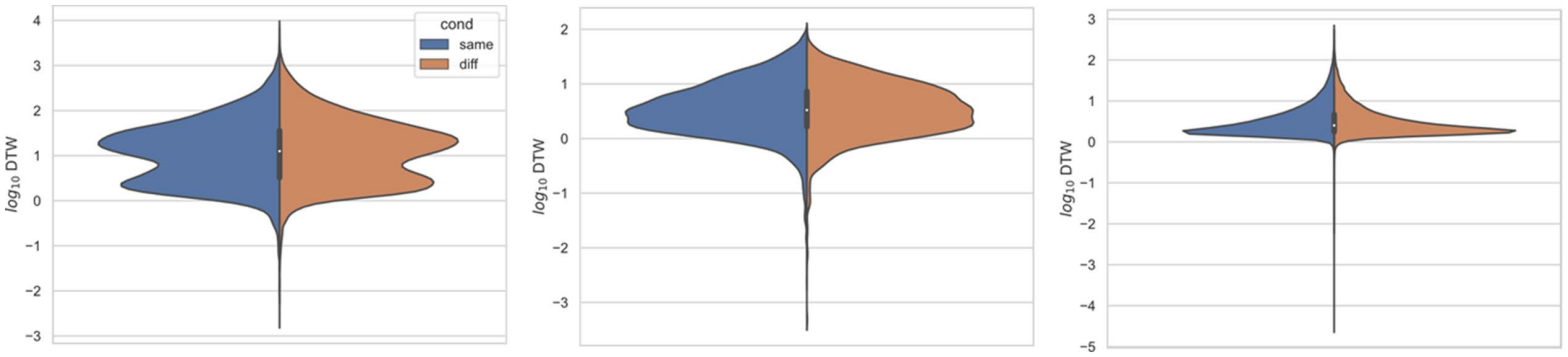
Comparing distributions for DTW matrix entries for neuron spike trains, depending on if the neurons compared were from the same vs. different electrode. Left: (Ito et al., 2018) (*n* = 25,542 same, *n* = 173,368 diff), middle: (Stout and Griffin, 2020) (*n* = 8,462 same, *n* = 10,186 diff), right: (Yang et al., 2022) (*n* = 125,580 same, *n* = 763,588 diff). For each study, Kolmogorov–Smirnov tests demonstrated significant (*p* < 0.001) differences between same vs. different electrode data.

**Figure 4:**
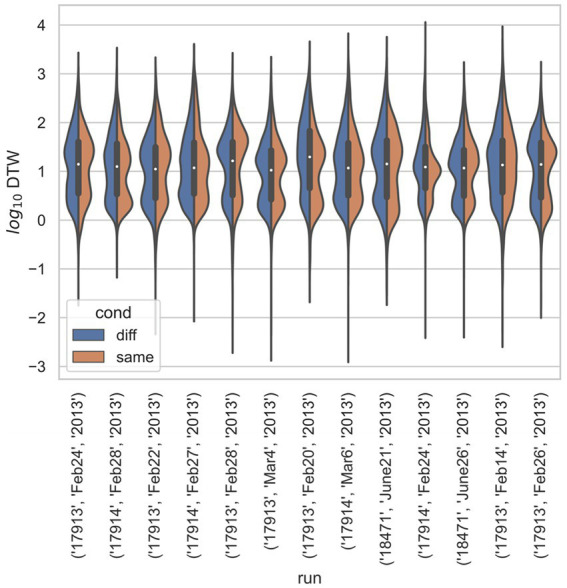
Stratified comparison of distributions for DTW matrix entries for neuron spike trains, for Ito et al. (2018), depending on if the neurons compared were from the same vs. different electrode. For all 13 runs, Kolmogorov–Smirnov tests demonstrated significant (*p* < 0.001) differences between the two populations.

**Figure 5:**
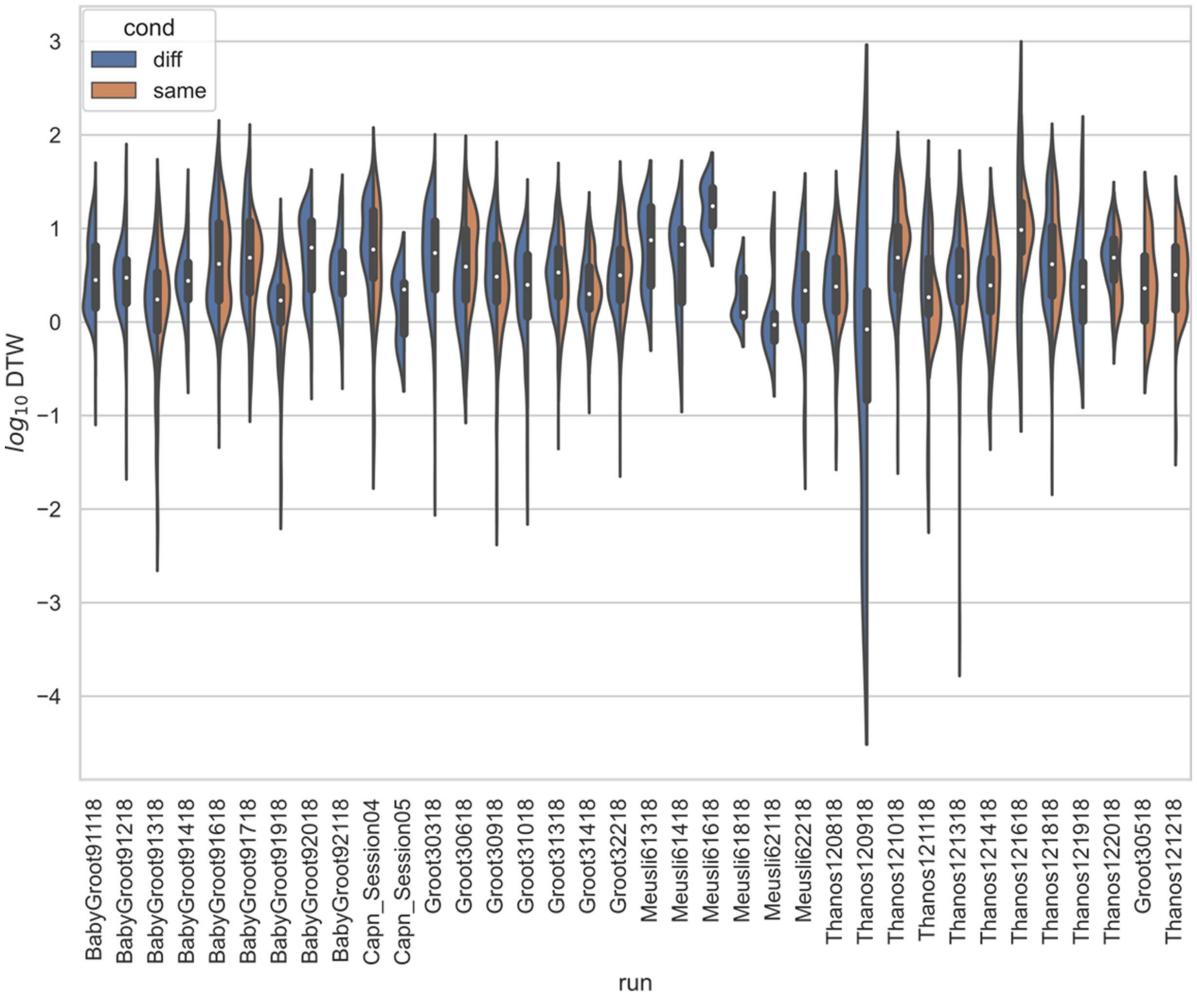
Stratified comparison of distributions for DTW matrix entries for neuron spike trains, for Stout and Griffin (2020), depending on if the neurons compared were from the same vs. different electrode. For all 34 runs with non-empty groups of same vs. diff, Kolmogorov–Smirnov tests demonstrated significant (*p* < 0.001, with the exception of run Thanos121618, for which *p* = 0.007) differences between the two populations.

**Figure 6:**
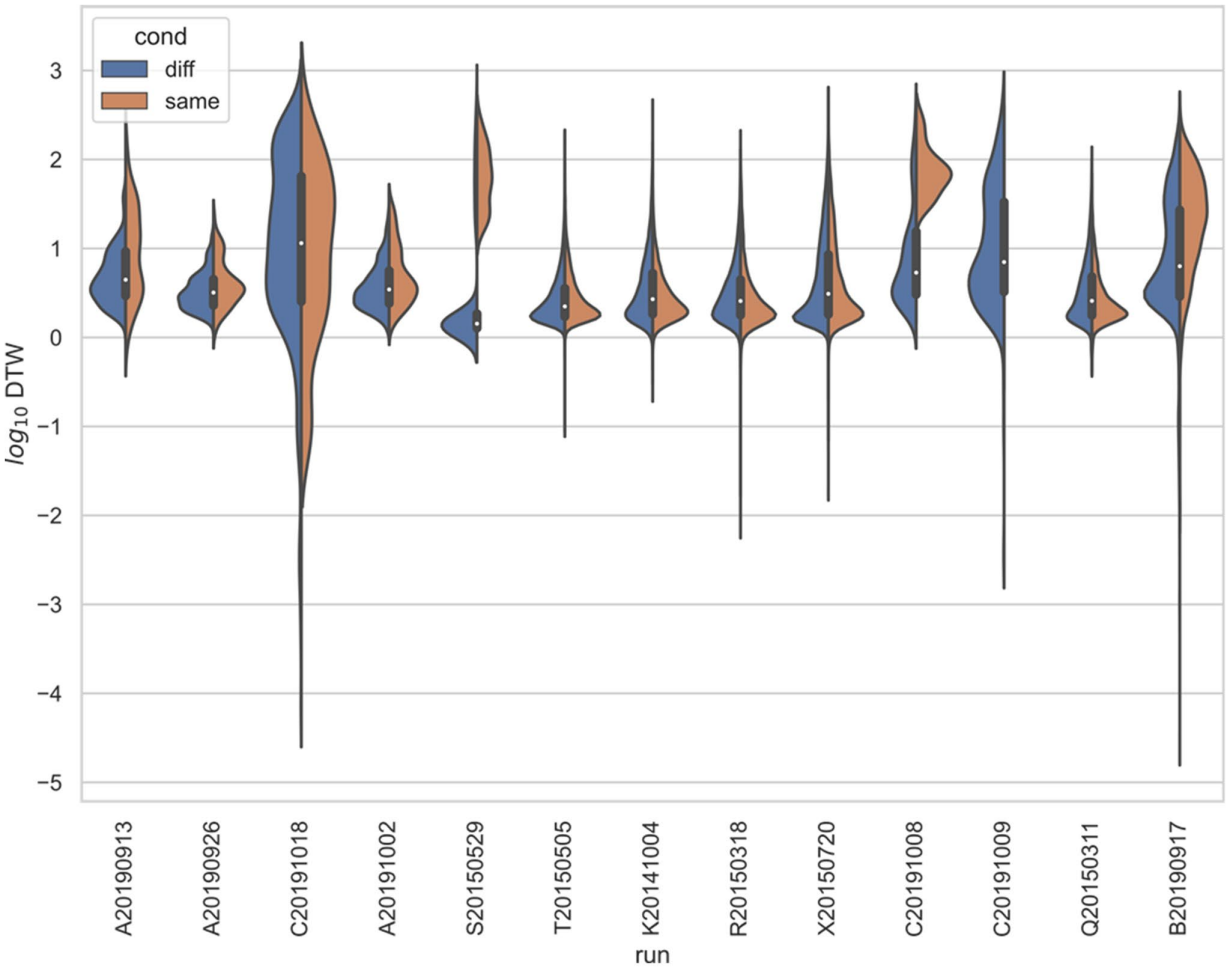
Stratified comparison of distributions for DTW matrix entries for neuron spike trains, for Yang et al. (2022), depending on if the neurons compared were from the same vs. different electrode. For all 13 runs, Kolmogorov–Smirnov tests demonstrated significant (*p* < 0.001) differences between the two populations.

In all three studies, the *d*_crit_ between neurons recorded from the same electrode versus from different electrodes were statistically significantly different (*p* < 0.001) by both the *t*-test and KS-test (Figures 3–6), however, this classification was useless as a predictor or regressor (Lo et al., 2015). For example, using *d*_cutoff_ as a classifier of *d*_crit_ for determining if two neurons were recorded from the same electrode vs. from different electrodes, the receiver operator characteristic (ROC) curve is essentially no different from random guessing (Supplementary Figure S4), with area under the curve (AUC) of 0.51. If we stratify data by session (Figures 4–6), however, the ROC curve and AUC vary for specific rats/sessions (Supplementary Figure S5), and in some cases demonstrate high predictive value (AUC).

**Figure 7:**
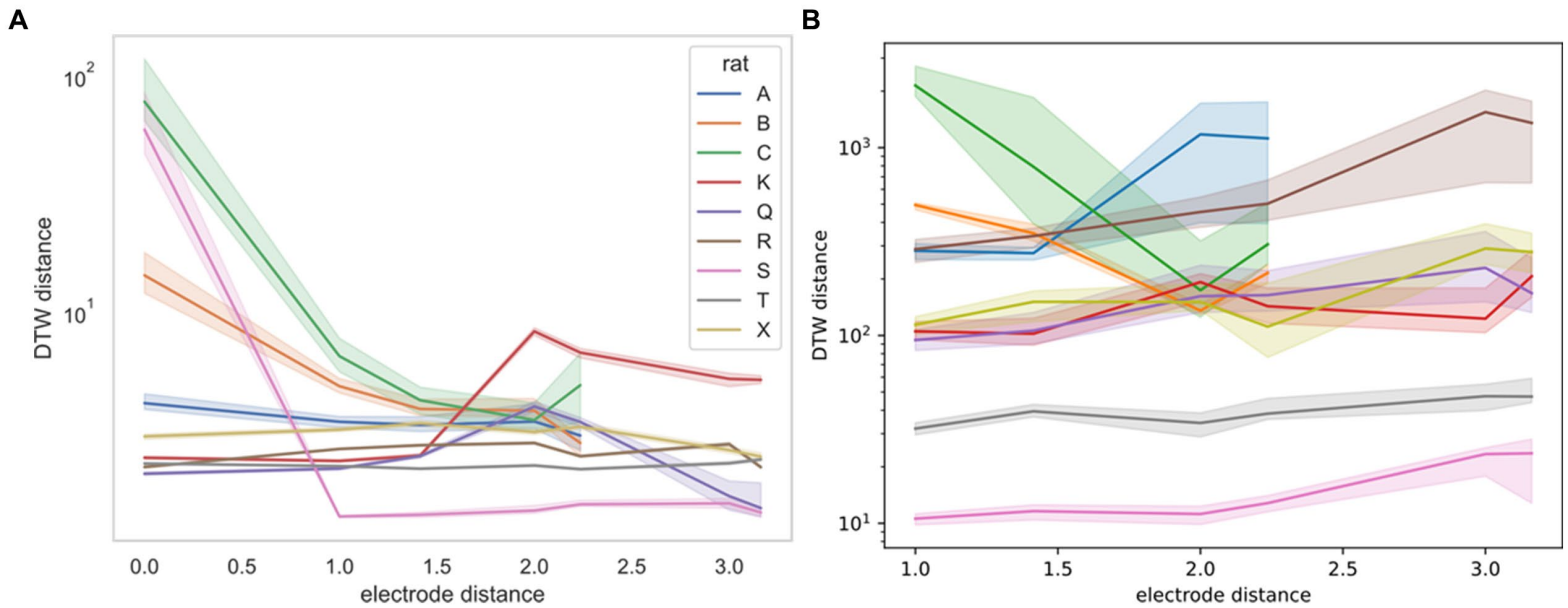
DTW distance for neuron spike trains (left, **A**) and LFPs (right, **B**) as a function of electrode distance (from Yang et al., 2022). The median DTW distance is plotted for each electrode distance, across all sessions for the indicated rat (rat A had 4 sessions, rat C had 3 sessions, and the remaining rats had a single session). 95% confidence intervals were estimated using 1,000 bootstrap samples. There is no consistent correlation between the DTW and electrode distance for either dataset, supporting the idea that there is no discernible spatial organization of neurons in the mPFC. Note that the DTW metric is different for the two datasets, with the distance for neuron spike trains reported as a time (ms) and the distance for LFPs reported as a voltage (V). Physical distance is reported in multiples of 0.25 mm.

For the Yang study (Yang et al., 2022), we were able to obtain the exact geometry of the electrode. Therefore, we were able to stratify DTW distances by the physical distance of the neurons measured (Figure 7). For this stratification, we were unable to determine a consistent trend. Rats B and C demonstrate decreased DTW distance (increased synchronicity) in both neural spike trains and LFPs, whereas the remaining rats demonstrated slightly increasing DTW distance (decreased synchronicity) in both, except for rat S. For rat S, however, the large decrease in DTW distance seen in the spike trains may not be captured in the LFPs, because there was no zero-distance comparison for LFPs, whereas multiple spike trains recorded from the same electrode could be thought of as having zero distance.

### Use the DTW matrix to correlate neural network firing with behavior

Finally, in an effort to identify potential correlations between neural network firing (i.e., as assessed by DTW matrix) and behavior, we used all the information available in the DTW matrix instead of characterizing network connectivity with a single number (*d*_crit_). We performed all pairwise comparisons for trials in the same rat to ask the question: given a single session, is there a difference in Pearson correlation coefficient of before vs. after depending on if we compare (1) before vs. after of the same trial to (2) before vs. after of different trials? We performed pairwise Mantel test comparisons among trials in the same session. We performed Kruskal-Wallis and Kolmogorov–Smirnov tests to determine if these distributions of the correlation coefficient and *p*-value were different (Supplementary Figures S2, S3; Supplementary Table S3). Note that the Pearson correlation coefficient is used as the overall signal from the data for use in the non-parametric tests, rather than as a standalone parametric test; similar non-parametric procedures relying on the Pearson correlation are used in protein fluorescence colocalization (Costes et al., 2004). Overall, we found that there was no significant difference trial-to-trial for the before vs. after DTW matrix correlation.

When we stratified by correctness (Supplementary Table S3), we did observe an effect for Kolmogorov–Smirnov tests on the Pearson correlation coefficient in 35.7% (5 out of 14) sessions. When we used *d*_crit_ alone, this proportion was 7.1% (1 out of 14). To evaluate if the KS-test/Mantel test combination was more powerful than the Kruskal-Wallis test on *d*_crit_, we performed a Boschloo exact test on the contingency table; it did not reach statistical significance (*p* = 0.159). We concluded that there was mixed evidence for the efficacy of Mantel tests for before vs. after DTW matrices in determining if a rat made the correct or incorrect choice at the T-intersection.

## Discussion

We demonstrated that the DTW distance and the computed parameter $d_{\mathrm{crit}}$ captured some of the mPFC neural network firing dynamics for both the spike trains and LFPs that were associated with T-maze task performance. We found weak evidence that the correctness of rat choice influences the firing dynamics (Supplementary Table S3). More importantly, we also found that significant heterogeneities exist among studies, animals, and sessions, as measured by DTW distance and $d_{\mathrm{crit}}$ . To demonstrate meaningful associations between behavior (e.g., T-maze task performance) and neural network activities (in the mPFC), the data and computed results must be consistent. We first address the question of statistical consistency through a theoretical discussion of the experiments and the assumptions made in neuroelectrophysiology.

### Theoretical limitations of current experiments in neuroelectrophysiology

There are two major issues with attempting to measure a specific population of neurons. First, precise surgical implantation of electrodes is required; second, enough neurons must be sampled to capture overall neural network dynamics that can be consistently associated with certain behaviors. These two issues synergize at our current level of intracranial electrode technology, complicating fine measurements of neurons. Although the rat brain is far smaller than a human brain [*ca.* 1 cm (Citron, 2012)], it still contains an estimated 21 million neurons (Korbo et al., 1990). Reproducible surgical implantation in a specific area of the rat brain such as the medial prefrontal cortex is therefore highly dependent on the fine-motor skill of the researcher, and there is no guarantee that the same neurons or circuits will be sampled.

In fact, in Ito et al. (2018) and Stout and Griffin (2020) the electrodes were purposefully adjusted after each session so that a different set of neurons would be sampled. Due to the lack of spatial organization of the prefrontal cortex, we postulate that the surgical sampling of neurons and circuits in the prefrontal cortex is essentially random, and that electrode adjustment similarly resamples the neurons and circuits being recorded. The only way to counteract the statistical effects of random sampling is through the measurement of a sufficiently large fraction of the neurons in the brain area under consideration. In the Methods, we illustrate these issues with a simple mathematical illustration (*Reproducibility of electrode recordings*, Figure 1D).

To provide a guide for researchers investigating the connection between neuroelectrophysiology and behavior, we asked what did each of the three studies do right and what could be improved? There are three experimental parameters we considered: *n*_neurons_ (the number of neurons recorded); *n*_network repeat_ (the number of times the same neural network is recorded); and *n*_rats_ (the number of biological replicates). We contend that all three of these parameters must be relatively high to make meaningful conclusions, especially if one wishes to use more powerful parametric statistical tests over the non-parametric tests we used in this study.

To have any hope of identifying the specific neural network firing patterns which lead to decision-making in the T-maze, we must consistently identify the neurons responsible (*n*_neurons_), the circuits responsible (*n*_network repeat_), and demonstrate that the conclusions generalize across organisms (*n*_rats_). By this simple reasoning, we contend that the three recent studies we have examined here do not allow us to draw conclusions, regardless of data analysis method (DTW or otherwise). Stout and Griffin (2020) recorded too few neurons (*n*_neurons_ ~ 1), and changed the neurons recorded across sessions (*n*_network repeat_ = 1) for *n*_rats_ = 5. Ito et al. (2018) recorded a moderate number of neurons (*n*_neurons_ ~ 20), but still changed the neurons recorded across sessions (*n*_network repeat_ = 1), with *n_rats_* = 3. Yang et al. (2022) recorded a moderate number of neurons (*n*_neurons_ ~ 20), and kept the same neurons recorded across sessions (*n*_network repeat_ = 5), but only performed these replicates for *n*_rats_ = 2; the other *n*_rats_ = 7 had (*n*_network repeat_ = 1). In short, larger electrodes (*n*_neurons_), more sessions per experimental condition (*n*_network repeat_), and more animals (*n*_rats_) are needed to achieve a robust and reproducible conclusion.

### Recommendations for reproducible neuroelectrophysiology

Our recommendation for electrode size (*n*_neurons_) depends on the number of neural clusters or circuits one wishes to investigate (Figure 1D), whereas *n*_network repeat_ and *n*_rats_ should be chosen according to traditional recommendations such that parametric statistical tests can be performed (de Winter, 2013; Curtis et al., 2015). For example, to estimate error bars for technical replicates or biological replicates, there should be at least *n = 5* independent technical trials or animals, respectively (Curtis et al., 2015). A useful control group would contain at least five animals, and each animal should be recorded on five separate occasions. Furthermore, if we wish to compare neural firing between experiments, then roughly the same neurons should be recorded, and any difference should be adjusted for in statistical tests.

### Efficacy of dynamic time warping in analyzing neuroelectrophysiology data

We now turn to the efficacy of DTW in potentially uncovering connections between neuron electrical activity and behavior. In our analysis, we demonstrated that the DTW distance and the computed parameter $d_{\mathrm{crit}}$ captured some of the mPFC neural network firing dynamics for both the spike trains and LFPs. We found weak evidence that the correctness of rat choice influences the firing dynamics (Supplementary Table S3). We expect, however, that many more experiments are needed to confirm the classification of the correctness of a decision based on mPFC activity. Our results indicated that whereas firing trials could be pooled across the same session, they could not be pooled across different sessions or studies (Supplementary Tables S1, S2). In terms of spatial dependence of firing synchronicity, we found that for specific rats and sessions, the classification of DTW distances for recordings taken from the same vs. different electrodes was both statistically significant (Figures 3, 4; Supplementary Figures S3, S4) and useful as a regressor (Supplementary Figure S5). For the Yang study (Yang et al., 2022), the spatial dependence of DTW distances was highly dependent on the specific rat sampled (Figure 7), providing evidence of the lack of spatial organization of the prefrontal cortex.

The difficulties we encountered in analyzing spike trains and LFPs can be addressed through changes to the experimental design. The number of neurons sampled is an important characteristic to consider, especially if only a few neurons were recorded and some recordings represented only a single neuron (e.g., Stout and Griffin, 2020). If the mPFC is composed of separate neural networks that perform specific, modular tasks, it is unlikely that recording a low number of neurons will provide meaningful, interpretable results. Since firing dynamics were consistent across a single session, we posit that multiple recordings of a single neuron population are the best approach to characterizing network firing behavior. The DTW spatial dependence remained consistent across multiple sessions for rats A and C (Figure 3), therefore we are reasonably confident that we recorded the same population of neurons across the sessions. The additional variation across sessions in Yang et al. (2022) might due to changes in the resting cognitive state of the rats. It was, however, not possible for us to distinguish consistently between variance due to noisy measurements and variance due to rat behavioral changes. It may be necessary to perform many control sessions to characterize in adequate detail all of the resting cognitive states of a specific rat. Crucially, recording a single control session as Yang et al. (2022) did for the seven other rats (B, K, Q, R, S, T, X) likely fails to detect variations in the resting state for a single rat. To complement the recording of many control sessions, recording additional rat behaviors may yield insight as to how the rat resting cognitive state influences the network firing dynamics. Because the electrodes were adjusted after each session in the Ito et al. (2018) and Stout and Griffin (2020) studies, the recording of only a single session per neuron population complicates the modeling that must be performed.

Recent advances in imaging and brain modeling provide hope that we may one day understand the relationship between neuron firing and consciousness. Whole brain connectomes have been mapped for simple organisms [*C. elegans* (Cook et al., 2019), *Ciona intestinalis* larvae (Ryan et al., 2016), and *Platynereis dumerilii* (Verasztó et al., 2020)], and recently all 3,000 neurons and 548,000 synapses of a fruit fly larva (*Drosophila melanogaster*) were mapped (Winding et al., 2023). Although mammalian brains are orders of magnitude larger than insect brains, whole-brain connectomes for rats may not be out of reach in the coming decades. These connectomes are essential for understanding cognition, but they provide only a static, anatomic reference upon which neural physiology and pathophysiology must be built. Our study highlights the limitation of the data analyses on spike trains and LFPs, implying that combining different modalities of data, such as connectome or imaging information, may be the next steps in investigating the function and dynamics of biological neural networks.

## Materials and methods

### Data set selection and the acquisition of additional rat recordings

Three published data sets were used for this study (Ito et al., 2018; Stout and Griffin, 2020; Yang et al., 2022). The correlation between the neural synchronicity and the choice behavior in the T-maze was not the focus of any of these datasets. These datasets, however, represent our best efforts in acquiring standardized data with homogeneous experimental conditions, i.e., (1) recent (2) rat (3) PFC recordings in the (4) T-maze task, and (5) providing both spike trains and LFPs. We set these five requirements to make the statistical tests as unbiased as possible so that the data can be assumed to be drawn from the same underlying distribution.

Besides the data we used from the three published studies, new data were collected from three additional male Sprague–Dawley rats (Charles River Laboratories). They weighed 300–350 g when received and were named as rats A, B, and C. The experimental procedure of T-maze alternation and neural recording followed published protocol (Yang et al., 2022). All animal care and surgical procedures were in accordance with the National Institutes of Health Guide for the Care and Use of Laboratory Animals and Penn State Hershey Animal Resources Program, and were reviewed and approved by the local IACUC.

### Dynamic time warping and statistical tests

Mathematical details on dynamic time warping, the Mantel test, and the Kolmogorov–Smirnov test are included in the Supplemental methods.

### Computation of *d*_crit_

Based on a DTW matrix *D*, we formed a graph *G* with nodes representing neurons and edges between nodes *i* and *j* with weight equal to the DTW distance between those neurons *D_ij_*. There is a certain value *d*_crit_ at which removing the edges from the graph with weight greater than *d*_crit_ results in the graph becoming disconnected. This value can be found by performing a binary search on *d*_crit_ between the minimum and maximum value in *D* such that depth-first search on the graph with removed edges no longer spans all the nodes (Cormen et al., 2009).

### Estimated number of neurons in the rat medial prefrontal cortex

Our goal here is to provide a reasonable estimate for the number of neurons that one must record to reconstruct the dynamics of the entire mPFC. We used existing estimates of neuron and synaptic density in the rat prefrontal cortex as starting points for our modeling. We set the total number of neurons in the rat brain (Korbo et al., 1990) to *N*_brain_ = 2.1 × 10 (Miyawaki et al., 2008) with volume (Hamezah et al., 2017) *V*_brain_ = 2,500 mm (Citron, 2012). We set the volume of the mPFC (Hamezah et al., 2017) to *V*_mPFC_ = 20 mm (Citron, 2012). Assuming a uniform distribution of neuron count throughout the brain, the estimated number of neurons in the mPFC was $N_{\mathrm{mPFC}}=N_{\mathrm{brain}}\times \frac{V_{\mathrm{mPFC}}}{V_{\mathrm{brain}}}=1.7\times 10^{5}$ . The assumption of a uniform distribution of neurons throughout the brain may not be entirely accurate. Neuron density can vary significantly across different regions of the brain due to functional specialization. The prefrontal cortex may have a different neuron density compared to other areas such as the sensory or motor cortices. This non-uniformity could lead to an over-or under-estimation of the actual number of neurons in the mPFC.

### Reproducibility of electrode recordings

Here, we discuss the problem of reproducibility in neuroelectrophysiology, specifically, the rodent spike train and local field potential recordings in the PFC during the T-maze alternation task. Because of the apparent lack of spatial organization in the mPFC, when electrodes are implanted, they uniformly sample the estimated *N*_mPFC_ = 10 (Hartline, 1969) neurons in the mPFC. Depending on how cooperative and synchronized neuron firing is, we assume that there is a certain number of neural clusters $n_{\mathrm{cluster}}$ for which the measurement of a single neuron sufficiently captures the behavior of the entire cluster, and that measurement of these clusters correlates with rat behavior. We also assumed that sampling is done with replacement, i.e., *n*_cluster_ < < *N*_mPFC_. For reproducibility, one must (1) record the same neural clusters across biological replicates and (2) identify or classify the neural clusters to show that findings in one animal generalize to other animals.

1. The first issue of capturing a certain percentage of the neural clusters in the rat brain corresponds to a classical problem in combinatorial probability known as the coupon collection problem. The mathematical question is: given *c = n_cluster_* categories, what is the expected number of samples S which needs to be drawn from those categories so that all categories are represented at least once? In the case of equal probabilities for drawing each category,

(2) $$S=c\cdot \overset{c}{\underset{i=1}{\sum }}\frac{1}{i}=cH_{c}\text{,}$$

where *H_c_* is the *cth* harmonic number. Alternatively, we may require that a certain number *1 < k < c* out of all the categories is represented (Ferrante and Frigo, 2012), so that Equation 2 is a special case of


(3) $$S\left(k\right)=c\left(H_{c}-H_{c-k}\right)=c\overset{k-1}{\underset{i=0}{\sum }}\frac{1}{c-i}\text{.}$$

In both cases, *S ~ c* log *c* asymptotically. We have plotted the expected number of clusters we may identify depending on the maximum number of neurons recorded from each of the studies (Figure 1D). In practice, it is necessary to first determine the number of neural clusters that exist and to choose neuron sample size that can adequately sample all the clusters with high probability.

1. The second issue of identifying neural clusters across different animals relies on the researcher’s ability to determine which of the *n_cluster_*! possible matchings is the correct matching between any two rats. To reduce the number of possibilities, one must rely either on prior information (such as typical firing rates or patterns of certain clusters) or on other measurements of network behavior that are invariant under permutations of nodes in the graph.

### Computational analysis and plotting

All computational analysis was carried out in Python 3.10 and R 4.1, on an M1 Max MacBook Pro with 64 GB RAM. For statistical tests: Shapiro–Wilk, Kruskal-Wallis, and ANOVA were carried out using pingouin 0.5.3 (Vallat, 2018), Mantel tests (The Scikit-Bio Development Team, 2023; Mantel, 1967) were carried out using scikit-bio 0.5.7 (The Scikit-Bio Development Team, 2023), and Kolmogorov–Smirnov and Boschloo exact tests were carried out using SciPy 1.8.1 (Virtanen et al., 2020). We implemented dynamic time warping (Vintsyuk, 1972) using Cython 0.29.32 (Behnel et al., 2011). We used the DataFrame structure from Pandas 1.5.1 (The Pandas Development Team, 2022) to organize our data, and we used seaborn 0.12.1 (Waskom, 2021) and Matplotlib 3.5.2 (Hunter, 2007) for plotting. We used NumPy 1.23.3 (Van Der Walt et al., 2011; Harris et al., 2020) for numerical array operations. We used ggpubr 0.6.0 (Kassambara, n.d.) for the balloon plots.

## Significance statement

The prefrontal cortex is important in decision-making, yet no robust method currently exists to correlate neuron firing in the PFC to behavior. We argue that existing experimental designs are ill-suited to addressing these scientific questions. To optimize the usage of existing data, we propose the use of dynamic time warping to analyze PFC neural electrical activity. We conclude that careful curation of experimental controls is needed to separate accurately true neural signals from noise.

## Data Availability

The original contributions presented in the study are included in the article/Supplementary material, further inquiries can be directed to the corresponding authors. All code and data which are necessary to reproduce the results in this work are posted on Zenodo (https://doi.org/10.5281/zenodo.7933346).
