# Supplementary material for: Quantifying network behavior in the rat prefrontal cortex
Source: Front Comput Neurosci. 2024 Aug 29;18:1293279. doi: 10.3389/fncom.2024.1293279 (PMC11390430; doi:10.3389/fncom.2024.1293279)
Supplement: Supplementary file 1 [file Data_Sheet_1.PDF]

Supplemental Material

Supplemental Methods 1
Dynamic time warping........ 1
Mantel test 2
Kolmogorov-Smirnov test........ 2
References 3
Supplemental Tables........ 4
Supplemental Table 1: Statistical tests for neuron spike trains. 4
Supplemental Table 2: Statistical tests for local field potentials. 6
Supplemental Table 3: Tests on changes in before vs after spike train DTW matrix correlation. 8
Supplemental Figures 9
Supplemental Figure 1:........ 9
Supplemental Figure 2:........ 10
Supplemental Figure 3:........ 11
Supplemental Figure 4:........ 12
Supplemental Figure 5:........ 13

Supplemental Methods

Dynamic time warping

Dynamic time warping (DTW) is a method for time series and sequence alignment[1]. Given two sequences of data $x_i = x(u_i)$ and $y_j = y(v_j)$ with lengths m and n respectively, DTW seeks to find a monotonically increasing function $w: u_i \to v_j$, such that $w(u_1) = v_1$, $w(u_m) = v_n$, such that each u_i is paired with a v_j and vice versa, and such that a measure of sequence distance $d(x, y)$ is minimized. This problem possesses an optimal substructure and therefore may be treated using dynamic programming for an efficient solution.

When the sequence distance is a sum of costs for each pair of values

$$d(x,y) = \sum_i d_s(x(u_i), y(w(u_i))) \quad (1)$$

we can express DTW as an algorithm on a matrix running in $O(mn)$ time.

Consider an alignment of x and y. The last symbols must be mapped to each other $x_m \leftrightarrow y_n$, and must be included in the total distance. There are three possibilities for how this alignment came about. First, y_n could be mapped to a previous $x_i, i < m$; second, y_n and x_m could be matched to each other, separately from the shorter sequences $\{y_1, \cdots, y_{n-1}\}$ and $\{x_1, \cdots, x_{m-1}\}$; third, x_m could be matched to a previous y_j, $j < n$. Denoting the distance of the alignment of the first i entries of x and the first j entries of y as $M(i, j)$, we have the recursion relation

$$M(m,n) = d_s(x_m, y_m) + \min\{M(m-1,n), M(m-1,n-1), M(m,n-1)\}\,. \quad (2)$$

The DTW method is shown in Algorithm 1.

We performed dynamic time warping on both the neuron spike trains and local field potentials (LFPs). Since the spike trains were all-or-none events and each entry of x and y represented an action potential at time t_i and t_j respectively, we used the distance function

$$d_{spike}\left(x(t_i), y(t_j)\right) = \left|t_i - t_j\right| \ . \tag{3}$$

For the local field potential, the measured quantity was the voltage, and we used the distance function

$$d_{LFP}\left(x(t_i), y(t_j)\right) = \left|x(t_i) - y(t_j)\right| \ . \tag{4}$$

For each pair of neuron spike trains for a given rat and each pair of LFP tracings, we computed the overall dynamic time warping distance according to Eq. (1), forming a distance matrix. One major caveat is that the DTW distance does not satisfy the triangle inequality and therefore is not a metric. However, DTW distances may be projected onto lower dimensions through principal coordinates analysis, classical multidimensional scaling, or clustering techniques for analysis.

Mantel test

To compare the neuron DTW distance matrices for spike trains and LFPs, we employed the Mantel test[2,3], a non-parametric test of matrix similarity. The idea of the Mantel test is to mitigate correlations among matrix entries by permuting the columns of one of the matrices. For a pair of matrices (M, N), the Mantel test computes a Pearson correlation coefficient ρ

$$\rho(M, N) = \frac{\text{cov}(M_{ij}, N_{ij})}{\sigma_M \sigma_N} = \frac{\sum_{ij}(M_{ij} - \bar{M})(N_{ij} - \bar{N})}{\sqrt{\sum_{ij}(M_{ij} - \bar{M})^2}\sqrt{\sum_{ij}(N_{ij} - \bar{N})^2}} \ ; \tag{5}$$

$\rho(M, N)$ is then compared to the distribution of $\rho_{perm}(M, N)$, in which Eq. (5) is computed for all permutations of the columns (or rows) of M (or N) while keeping the other matrix fixed. For large matrices, the permutations are sampled from the set of all permutations.

The location of $\rho(M, N)$ with respect to all the $\rho_{perm}(M, N)$ gives an estimate of the similarity of the matrices.

Kolmogorov-Smirnov test

To compare DTW matrices between rats, we used the Kolmogorov-Smirnov (KS) test[4,5] to compare distributions of the relative percentiles of the Pearson correlation coefficient computed from the Mantel test. The KS test is a parameter-free test which determines if samples drawn from two populations represent different underlying probability distributions.

First, the empirical distribution function (P_1 and P_2) of each population is estimated based on the samples,

$$P_k(x) = \sum_i \delta\left(x - s_{k,i}\right) \ , \tag{6}$$

where $\delta(x)$ is the Dirac delta function and $s_{k,i}$ is the ith sample from distribution k. Second, the empirical cumulative distribution function (F_1 and F_2) for each population is estimated as

$$F_k(x) = \int_{-\infty}^{x} P_k(t) \, \text{dt} \ . \tag{7}$$

Finally, the KS statistic is computed:

$$D = \sup_{x} \ |F_1(x) - F_2(x)| \ . \qquad (8)$$

For a confidence level α, the null hypothesis is rejected (i.e. the underlying probability distributions are different) if

$$D > \sqrt{-\log\left(\frac{\alpha}{2}\right) \cdot \left[\frac{1+\frac{m}{n}}{2m}\right]} \ . \qquad (9)$$

where m and n are the sample sizes.

References

1. Vintsyuk, T. K. Speech discrimination by dynamic programming. *Cybernetics* **4**, 52–57 (1972).
2. Mantel, N. The Detection of Disease Clustering and a Generalized Regression Approach. *Cancer Res* **27**, 209–220 (1967).
3. Vallat, R. Pingouin: statistics in Python. *J Open Source Softw* **3**, 1026 (2018).
4. Kolmogorov, A. N. Sulla determinazione empirica di una legge di distribuzione. *Giornale dell'Istituto Italiano degli Attuari* 83–91 (1933).
5. Virtanen, P. *et al.* SciPy 1.0: fundamental algorithms for scientific computing in Python. *Nat Methods* **17**, 261–272 (2020).

Supplemental Tables

Supplemental Table 1: Statistical tests for neuron spike trains.

Categorical variable	Null hypothesis (H_0)	Alternative hypothesis (H_A)	Test	Additional restrictions/stratification	Fraction of tests with p < 0.05 (number out of total)
None	The d_{crit} are normally distributed for each run.	The d_{crit} are NOT normally distributed for each run.	Shapiro-Wilk	None	0.967 (60 out of 62 runs)
				correctness = true	0.967 (60 out of 62 runs)
				correctness = false	0.967 (60 out of 62 runs)
				timing = before	0.967 (60 out of 62 runs)
				timing = after	0.967 (60 out of 62 runs)
				timing = before AND correctness = true	0.967 (60 out of 62 runs)
				timing = before AND correctness = false	0.967 (60 out of 62 runs)
				timing = after AND correctness = true	0.967 (60 out of 62 runs)
Events in a single run	The d_{crit} for each event does not vary across a run.	At least one event in the run has a significantly different d_{crit} from the others.	Kruskal-Wallis	None	0.048 (3 out of 62 runs)
				correctness = true	0.048 (3 out of 62 runs)
				correctness = false	0.048 (3 out of 62 runs)
				timing = before	0.048 (3 out of 62 runs)
				timing = after	0.048 (3 out of 62 runs)
				timing = before AND correctness = true	0.048 (3 out of 62 runs)
				timing = before AND correctness = false	0.048 (3 out of 62 runs)
				timing = after AND correctness = true	0.048 (3 out of 62 runs)
				timing = after AND correctness = false	0.048 (3 out of 62 runs)
			ANOVA	None	0.112 (7 out of 62 runs)
				correctness = true	0.112 (7 out of 62 runs)
				correctness = false	0.112 (7 out of 62 runs)
				timing = before	0.112 (7 out of 62 runs)
				timing = after	0.112 (7 out of 62 runs)
				timing = before AND correctness = true	0.112 (7 out of 62 runs)
				timing = before AND correctness = false	0.112 (7 out of 62 runs)
				timing = after AND correctness = true	0.112 (7 out of 62 runs)
				timing = after AND correctness = false	0.112 (7 out of 62 runs)
Events across all runs for a rat	The d_{crit} for each event does not vary	At least one event across all runs for the	Kruskal-Wallis	None	0.700 (7 out of 10 rats)
				correctness = true	0.700 (7 out of 10 rats)
				correctness = false	0.700 (7 out of 10 rats)

	across all runs for a rat.	rat has a significantly different d_{crit} from the others.		timing = before	0.700 (7 out of 10 rats)
				timing = after	0.700 (7 out of 10 rats)
				timing = before AND correctness = true	0.700 (7 out of 10 rats)
				timing = before AND correctness = false	0.700 (7 out of 10 rats)
				timing = after AND correctness = true	0.700 (7 out of 10 rats)
Events across all events for all rats in the study	The population of d_{crit} sampled is independent of rat in the study.	At least one event across all rats in the study has a significantly different d_{crit} from the others.	Kruskal-Wallis	None	0.667 (2 out of 3 studies)
				correctness = true	0.667 (2 out of 3 studies)
				correctness = false	0.667 (2 out of 3 studies)
				timing = before	0.667 (2 out of 3 studies)
				timing = after	0.667 (2 out of 3 studies)
				timing = before AND correctness = true	0.667 (2 out of 3 studies)
				timing = before AND correctness = false	0.667 (2 out of 3 studies)
				timing = after AND correctness = true	0.667 (2 out of 3 studies)

Supplemental Table 2: Statistical tests for local field potentials.

**Categorical variable**	**Null hypothesis (H_0)**	**Alternative hypothesis (H_A)**	**Test**	**Additional restrictions**	**Fraction of tests with p < 0.05 (number out of total)**
None	The d_{crit} are normally distributed for each run.	The d_{crit} are NOT normally distributed for each run.	Shapiro-Wilk	None	0.800 (20 out of 25 runs)
				correctness = true	0.800 (20 out of 25 runs)
				correctness = false	0.800 (20 out of 25 runs)
				timing = before	0.800 (20 out of 25 runs)
				timing = after	0.800 (20 out of 25 runs)
				timing = before AND correctness = true	0.800 (20 out of 25 runs)
				timing = before AND correctness = false	0.800 (20 out of 25 runs)
				timing = after AND correctness = true	0.800 (20 out of 25 runs)
Events in a single run	The d_{crit} for each event does not vary across a run.	At least one event in the run has a significantly different d_{crit} from the others.	Kruskal-Wallis	None	0.120 (3 out of 25 runs)
				correctness = true	0.120 (3 out of 25 runs)
				correctness = false	0.120 (3 out of 25 runs)
				timing = before	0.120 (3 out of 25 runs)
				timing = after	0.120 (3 out of 25 runs)
				timing = before AND correctness = true	0.120 (3 out of 25 runs)
				timing = before AND correctness = false	0.120 (3 out of 25 runs)
				timing = after AND correctness = true	0.120 (3 out of 25 runs)
				timing = after AND correctness = false	0.120 (3 out of 25 runs)
			ANOVA	None	0.200 (5 out of 25 runs)
				correctness = true	0.200 (5 out of 25 runs)
				correctness = false	0.200 (5 out of 25 runs)
				timing = before	0.200 (5 out of 25 runs)
				timing = after	0.200 (5 out of 25 runs)
				timing = before AND correctness = true	0.200 (5 out of 25 runs)
				timing = before AND correctness = false	0.200 (5 out of 25 runs)
				timing = after AND correctness = true	0.200 (5 out of 25 runs)
				timing = after AND correctness = false	0.200 (5 out of 25 runs)
Events across all runs for a rat	The d_{crit} for each event does not vary across all runs for a rat.	At least one event across all runs for the rat has a significantly different d_{crit} from the others.	Kruskal-Wallis	None	0.800 (4 out of 5 rats)
				correctness = true	0.800 (4 out of 5 rats)
				correctness = false	0.800 (4 out of 5 rats)
				timing = before	0.800 (4 out of 5 rats)
				timing = after	0.800 (4 out of 5 rats)
				timing = before AND correctness = true	0.800 (4 out of 5 rats)

				timing = before AND correctness = false	0.800 (4 out of 5 rats)
				timing = after AND correctness = true	0.800 (4 out of 5 rats)
Events across a study	The population of d_{crit} sampled is independent of rat in the study.	At least one event across all rats in the study has a significantly different d_{crit} from the others.	Kruskal-Wallis	None	1.000 (2 out of 2 studies)
				correctness = true	1.000 (2 out of 2 studies)
				correctness = false	1.000 (2 out of 2 studies)
				timing = before	1.000 (2 out of 2 studies)
				timing = after	1.000 (2 out of 2 studies)
				timing = before AND correctness = true	1.000 (2 out of 2 studies)
				timing = before AND correctness = false	1.000 (2 out of 2 studies)
				timing = after AND correctness = true	1.000 (2 out of 2 studies)

Supplemental Table 3: Tests on changes in before vs after spike train DTW matrix correlation.

**Categorical variable**	**Null hypothesis (H_0)**	**Alternative hypothesis (H_A)**	**Dependent variable**	**Test**	**Fraction of tests with $p < 0.05$ (number out of total)**
Same vs different event in a single run	There is no difference for Mantel tests of before vs after depending on if the before and after matrices are taken from the same vs different events in a single run.	There is a difference in Mantel test results depending on if the before and after matrices are taken from the same vs different events in a single run.	Pearson correlation coefficient (r)	Kruskal-Wallis	0.045 (2 out of 44)
				Kolmogorov-Smirnov	0.045 (2 out of 44)
			Mantel test p-value	Kruskal-Wallis	0.069 (3 out of 43)
				Kolmogorov-Smirnov	0.045 (2 out of 44)
Correct vs incorrect events in a single run	There is no difference for Mantel tests of before vs after depending on if the before and after matrices are taken from the correct vs incorrect events in a single run.	There is a difference in Mantel test results depending on if the before and after matrices are taken from the correct vs incorrect events in a single run.	Pearson correlation coefficient (r)	Kruskal-Wallis	0.000 (0 out of 13)
				Kolmogorov-Smirnov	0.357 (5 out of 14)
			Mantel test p-value	Kruskal-Wallis	0.000 (0 out of 9)
				Kolmogorov-Smirnov	0.214 (3 out of 14)
Correct vs incorrect events in a single run	d_{crit} is independent of correctness of events in a single run.	d_{crit} depends on correctness of events in a single run.	d_{crit}	Kruskal-Wallis	0.071 (1 out of 14)

Supplemental Figures

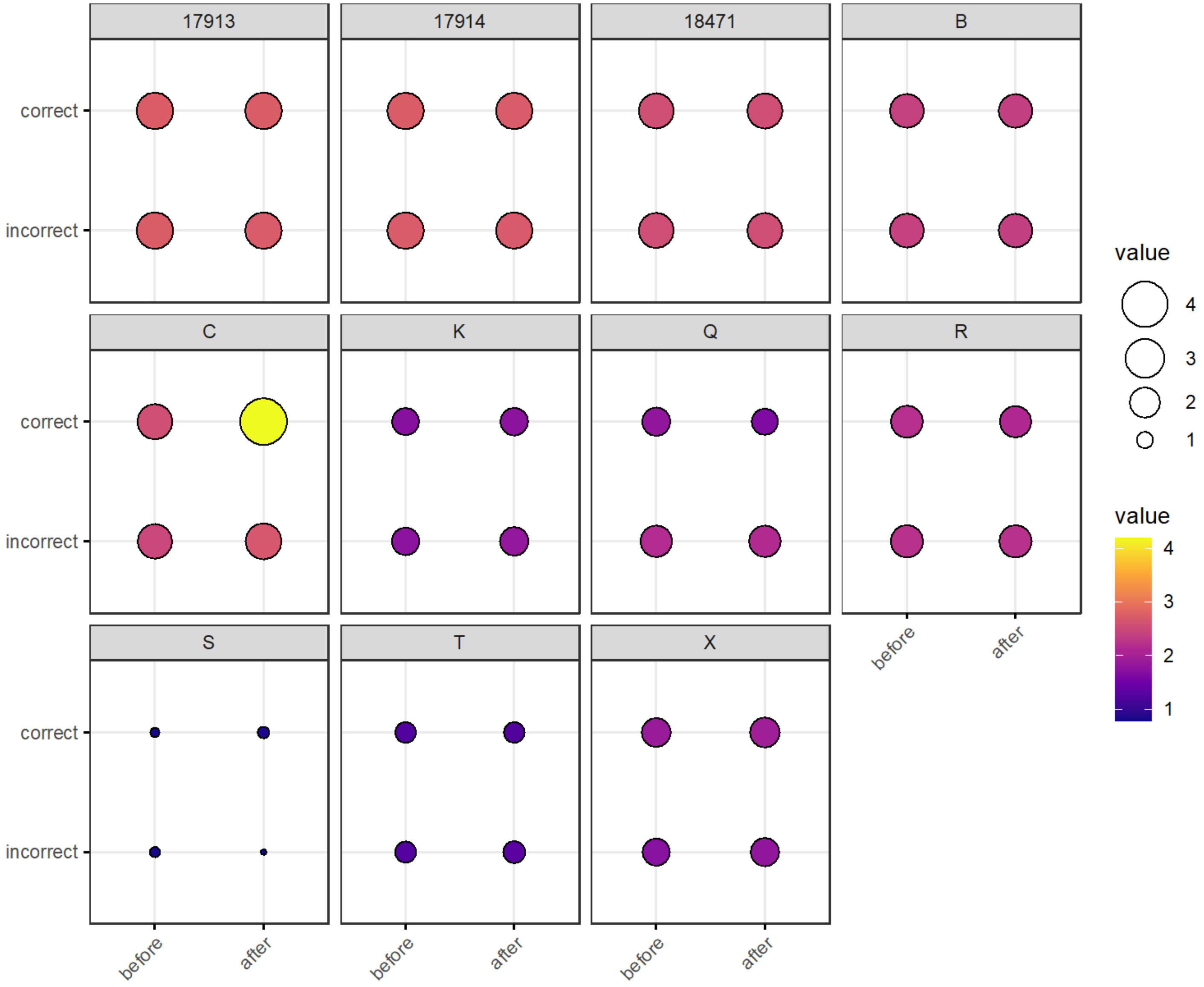

Supplemental Figure 1:

Balloon plot of local field potential d_{crit}, grouped by rat, correctness of T-arm choice, and timing with respect to T-arm choice. Each box represents a single rat. The x-axis represents timing (before vs after the rat visits the T-maze intersection) and the y-axis represents correctness of T-arm taking (True vs False). The size and color of each marker represents $\log_{10} d_{\text{crit}}$. For rats with multiple trials, we took the mean of $\log_{10} d_{\text{crit}}$. The scale of $\log_{10} d_{\text{crit}}$ is larger than that for the spike trains, visualized in Figure 1. The balloon plot visually appears more homogeneous. Local field potentials were not recorded in the Stout 2020 study.

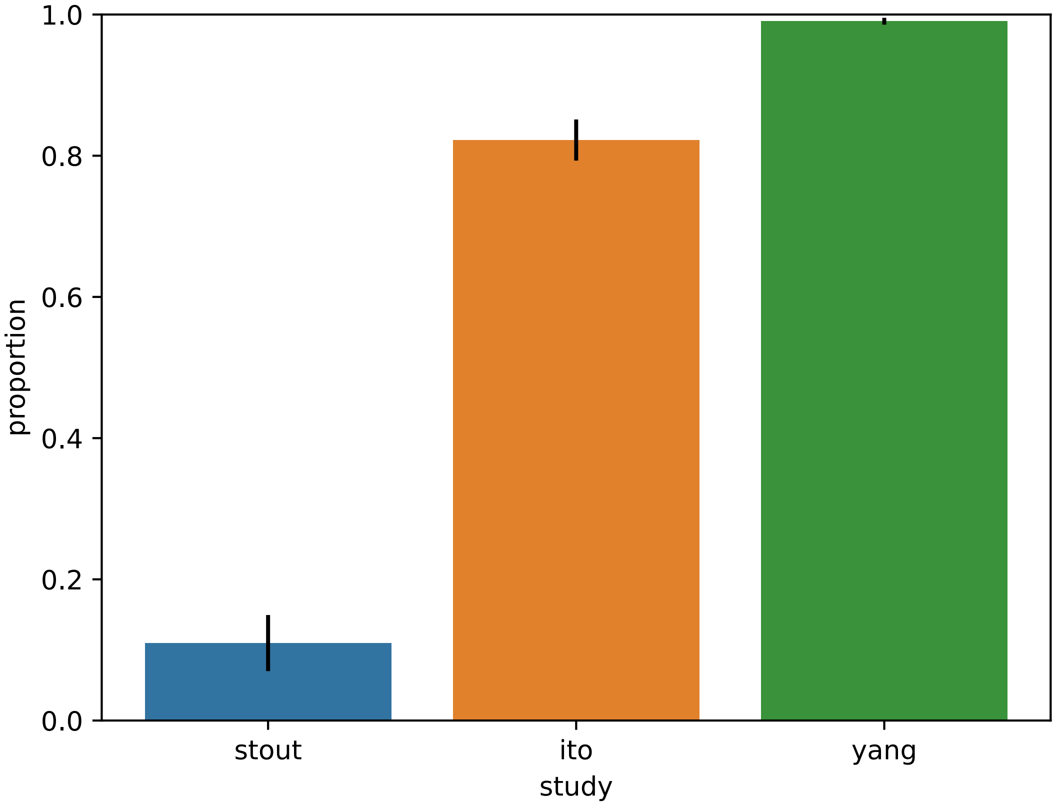

Supplemental Figure 2:

The proportion of Mantel tests which were significant ($p < 0.05$), stratified by study. A significant Mantel test indicates significant correlation between the two spike train DTW matrices which were compared, and therefore an indication of the consistency of recordings. We plotted 95% confidence intervals using the Agresti-Coull interval. As expected, the Yang 2022 study was the most consistent, since the electrodes were fixed across events and runs. In contrast, recordings from Stout 2020 were limited in the number of neurons recorded, and the electrodes were also not fixed, resulting in a low proportion of significant Mantel tests.

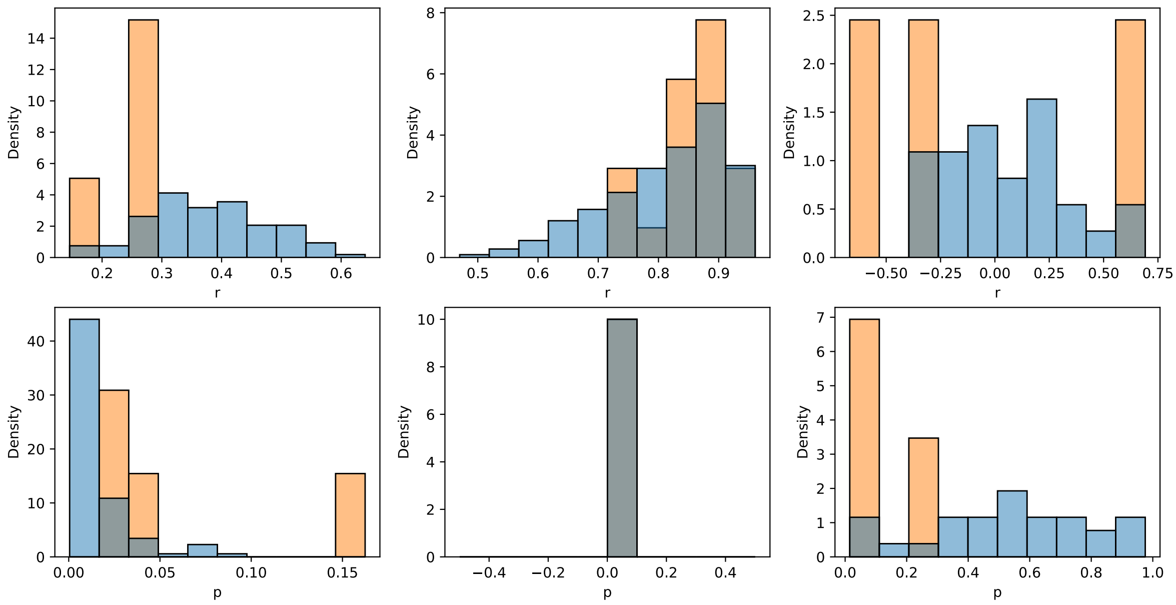

Supplemental Figure 3:

Mantel test correlation and p-value distribution for events across three runs, stratified by comparison. From left to right, the columns represent runs: (1) 17914, Feb 24, 2013; (2) Q, Mar 11, 2015; and (3) Groot, Mar 13, 2018. The top row shows Pearson correlation coefficient distributions, and the bottom row shows p-value distributions. The blue represents before vs after comparisons for different events in the same run, whereas the orange represents before vs after comparisons for the same event. Using Kruskal-Wallis tests, (1) and (2) had different distributions of correlation coefficient ($p < 0.05$), while (1) and (3) had different distributions of p-values ($p < 0.05$).

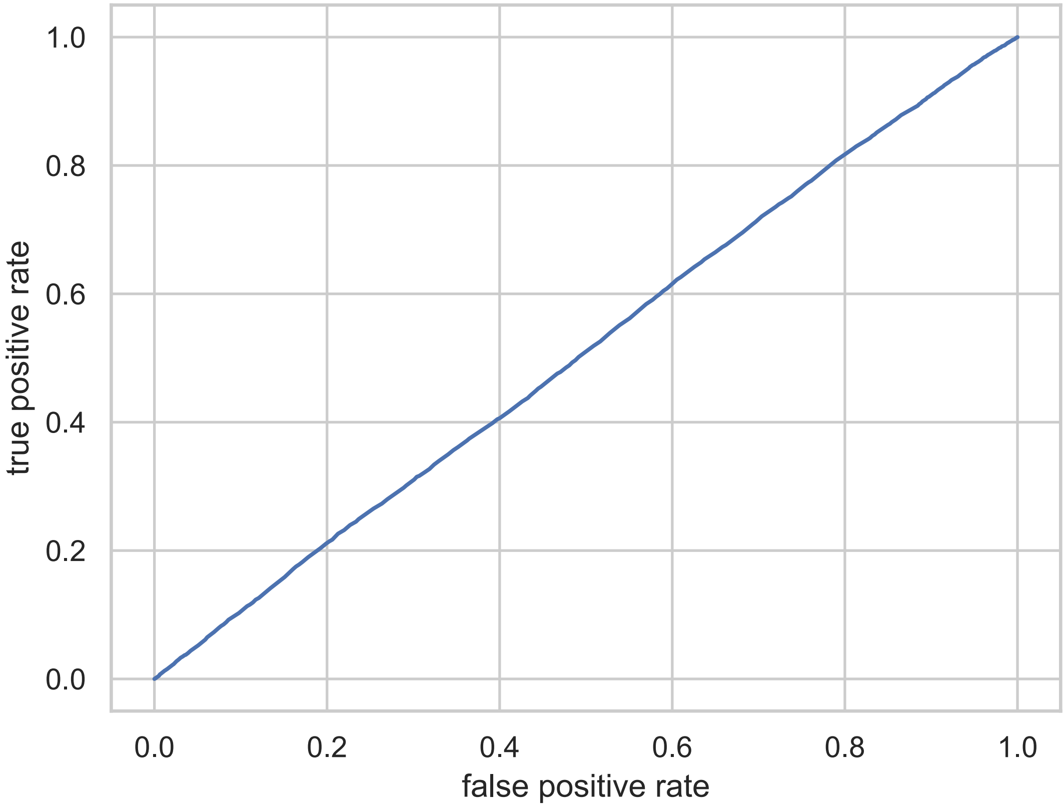

Supplemental Figure 4:

ROC curve for spatial data from Ito 2018, using cutoffs for the DTW matrix value as a predictor for the categorical variable same vs different electrode. The AUC is 0.51, indicating limited predictive value.

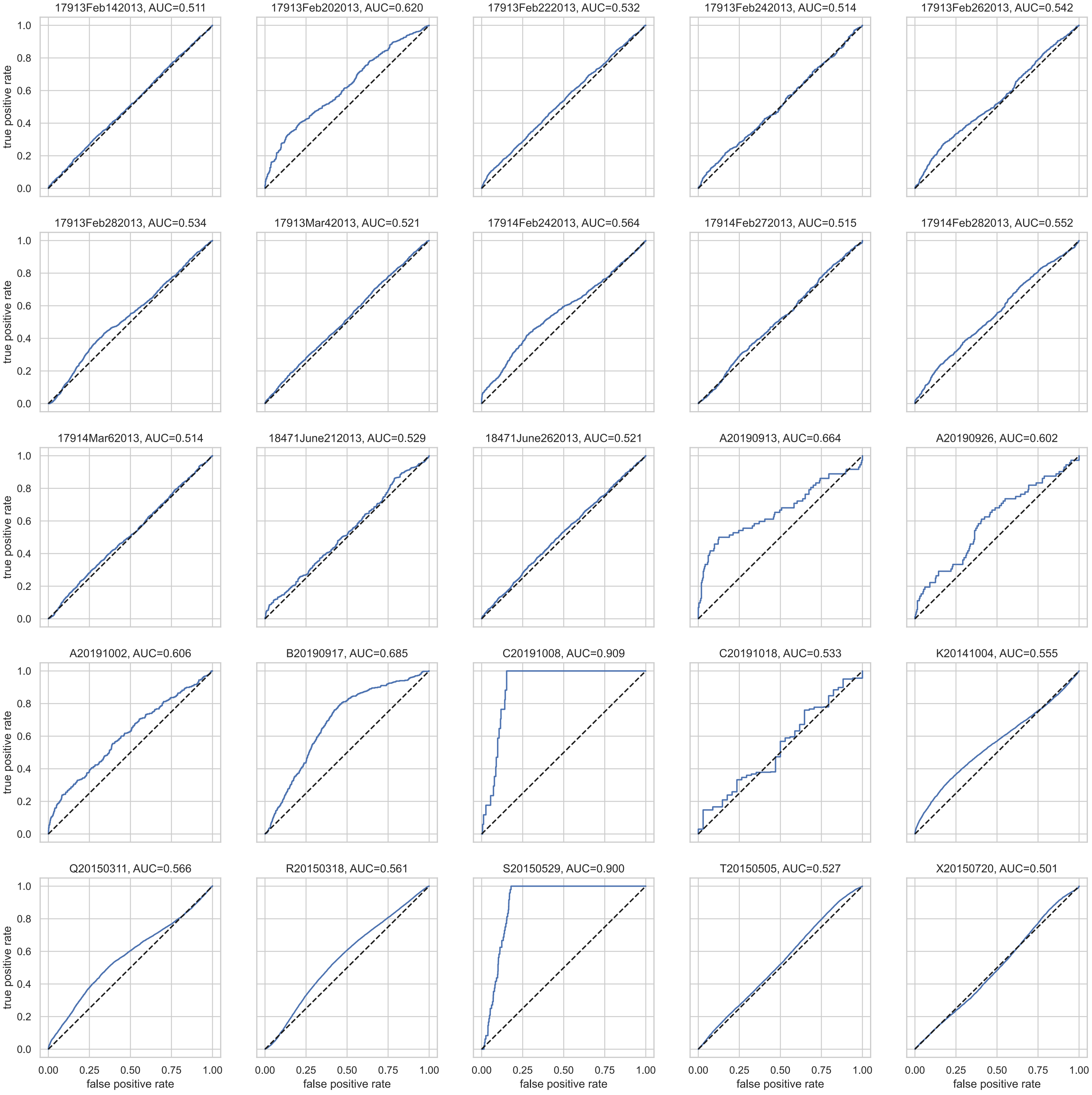

Supplemental Figure 5:

ROCs for each of the Ito 2018 and Yang 2022 runs, using a certain value of DTW distance as a cutoff for classifying the reading as from the same vs different electrodes. We omit Stout here because the number of DTW entries per matrix was not comparable. There is significant variability in AUC across the runs. The dotted line indicates random guessing; when the ROC dips below the dotted line, DTW matrix entries are consistently being classified into the wrong class.
